# Optically tuned terahertz modulator based on annealed multilayer MoS_2_

**DOI:** 10.1038/srep22899

**Published:** 2016-03-08

**Authors:** Yapeng Cao, Sheng Gan, Zhaoxin Geng, Jian Liu, Yuping Yang, Qiaoling Bao, Hongda Chen

**Affiliations:** 1 State Key Laboratory of Integrated Optoelectronics, Institute of Semiconductors, Chinese Academy of Sciences, Beijing, 100083, China; 2 State Key Laboratory of Superlattices and Microstructures, Institute of Semiconductors, Chinese Academy of Sciences, Beijing, 100083, China; 3 Institute of Functional Nano and Soft Materials (FUNSOM), Soochow University, Suzhou 215123, China; 4 School of Information Engineering, Minzu University of China, Beijing 100081, China; 5 College of Science, Minzu University of China, Beijing 100081, China

## Abstract

Controlling the propagation properties of terahertz waves is very important in terahertz technologies applied in high-speed communication. Therefore a new-type optically tuned terahertz modulator based on multilayer-MoS_2_ and silicon is experimentally demonstrated. The terahertz transmission could be significantly modulated by changing the power of the pumping laser. With an annealing treatment as a p-doping method, MoS_2_ on silicon demonstrates a triple enhancement of terahertz modulation depth compared with the bare silicon. This MoS_2_-based device even exhibited much higher modulation efficiency than the graphene-based device. We also analyzed the mechanism of the modulation enhancement originated from annealed MoS_2_, and found that it is different from that of graphene-based device. The unique optical modulating properties of the device exhibit tremendous promise for applications in terahertz switch.

In the last decade, terahertz (THz) waves, whose wavelength is 100–1000 μm, have aroused researchers’ interest because of its unique properties. Recently, THz technology has been applied in imaging^1^, bio-sensing^2^ and communication^3^,^4^. Meanwhile, a great of progresses has been made in THz devices such as THz sources^5^,^6^, detectors^7^,^8^, and modulators. Among these devices, THz modulators are very important in THz communication. In previous studies, different types of THz wave modulators have been reported, including devices based on GaAs^9^, silicon^10^, metamaterials^11^,^12^,^13^,^14^,^15^,^16^, silicon on sapphire (SOS)^17^, germanium^18^ and graphene^18^,^19^,^20^,^21^,^22^,^23^,^24^. As THz waves can be absorbed by a conductor, the basic way to modulate THz waves is to tune the conductivity of a semiconductor with an external voltage signal or a pumping light. Though both electrically and optically tuned modulators are practicable, the modulation speed of an optically tuned device is usually faster than that of an electrically tuned one^25^, as the latter is limited by the device charging time of depletion capacitance at the Schottky diode formed by the contact of metal and semiconductor^26^. For optically tuned devices, the modulation speed is mainly correlated with the lifetime of the photogenerated free carriers. Most of the optically tuned devices reported in literatures use femtosecond lasers as optical pumping sources to provide high power pulses so that massive free carriers can be excited during the THz probing. Switching time less than several nanoseconds and modulation depth of more than 70% have been demonstrated in those experiments^27^. However, it is more practicable in applications to use a continuous wave (CW) laser instead of femtosecond laser to modulate the pumping light with a particular signal. The disadvantage for CW lasers lies that its transient beam power is much lower than that of a femtosecond laser. Hence the concentration of free carriers in the device is much smaller and the change of conductivity may be not enough to realize a large THz modulation depth. One solution to improve the THz modulation efficiency is to use lightly doped silicon or materials with long carrier lifetime, such as germanium^18^. Meanwhile, a longer lifetime of free carriers means a slower modulation speed. One has to make a balance between the modulation depth and speed. Another solution is to introduce new materials into the device, to enhance the modulation effect of THz waves. Recently Rahm *et al*.^22^ has demonstrated a THz modulator based on optically tuned graphene-silicon system. A significant enhancement in THz transmission was induced by graphene due to its high mobility.

Graphene is just one of the numerous magical two-dimensional (2D) materials. Among these 2D materials, molybdenum disulfide (MoS_2_) shows unique properties^28^,^29^. The band gap of MoS_2_ depends on the number of its molecule layers. Single-layer MoS_2_ has a direct band gap of ~1.8 eV, while multi-layer MoS_2_ has an indirect band gap of 1.2 eV^30^. MoS_2_-based devices such as photodetectors and transisters have already been reported^31^,^32^,^33^. The optical and electric properties of MoS_2_-based devices can be tuned by defect engineering and doping^34^. It has been demonstrated that p-doped MoS_2_ showed great enhancement in photoluminescence intensity^35^,^36^. The doping process can be realized by a simple process of annealing in air or by mild O_2_ plasma irradiation^37^,^38^,^39^. Recently, another property of MoS_2_ is demonstrated in THz range^40^. MoS_2_ was observed to have ultrafast photoconductivity responses of less than 1 ps. This result indicates that MoS_2_ is suitable for application in high-speed THz devices. However, to our knowledge, THz modulators based on MoS_2_ have not been seen until now. In this paper, we demonstrate an optically tuned terahertz modulator based on multilayer MoS_2_ with an annealing treatment. It is demonstrated that annealed MoS_2_ can significantly enhance the THz modulation depth of silicon under a CW pumping laser. The modulation efficiency of our device is even higher than that of graphene-based THz modulators. Therefore our work provides a new method to realize an effective THz wave modulator.

## Results

**Figure 1:**
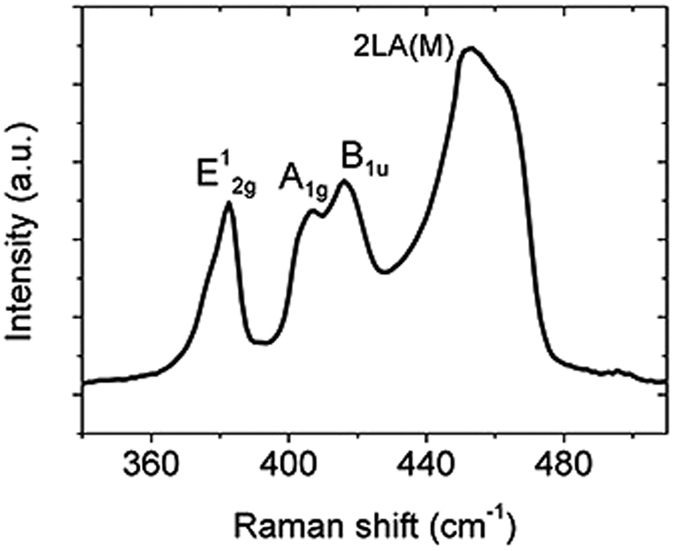
The Raman spectrum of multilayer MoS_2_ sample on a SiO_2_/Si substrate.

The sample of multilayer MoS_2_ was grown on a SiO_2_/Si substrate by chemical vapor deposition (CVD). It was then transferred to a clean high-resistivity silicon substrate (1 mm thick, resistivity *ρ* > 5000 Ω cm, *N*-type). The area of CVD MoS_2_ on silicon substrate is about 1 cm^2^. The multilayer MoS_2_ is verified by Raman spectroscopy. The Raman spectrum excited by 632 nm line is shown in Fig. 1a. Multiple peaks are observed including the *E*^*1*^_*2g*_ (383 cm^−1^), *A*_1g_ (406.6 cm^−1^), *B*_1u_ (416 cm^−1^) and 2LA(M) (453 cm^−1^) peaks, agreeing with results reported in ref. 28. The position difference of *E*^1^_2g_ and *A*_1g_ peaks is Δ = 23.6 cm^−1^, indicating that the MoS_2_ is multilayer.

The terahertz transmission spectra of the samples were measured using terahertz time-domain spectroscopy (THz TDS) system. A CW laser (808 nm) was used as an optical pumping source to excite photogenerated carriers in the samples. The diameter of the spot size of THz beam is about 8 mm and the beam diameter of the CW laser is about 1.5 cm. The broadband THz transmission spectra of MoS_2_ on silicon sample (MOS) were measured under different optical pump power. Transmission spectra of an identical bare high-resistivity silicon sample under the same conditions were measured as a reference. A sample with single-layer graphene on silicon (GOS) was also measured as a comparison.

**Figure 2:**
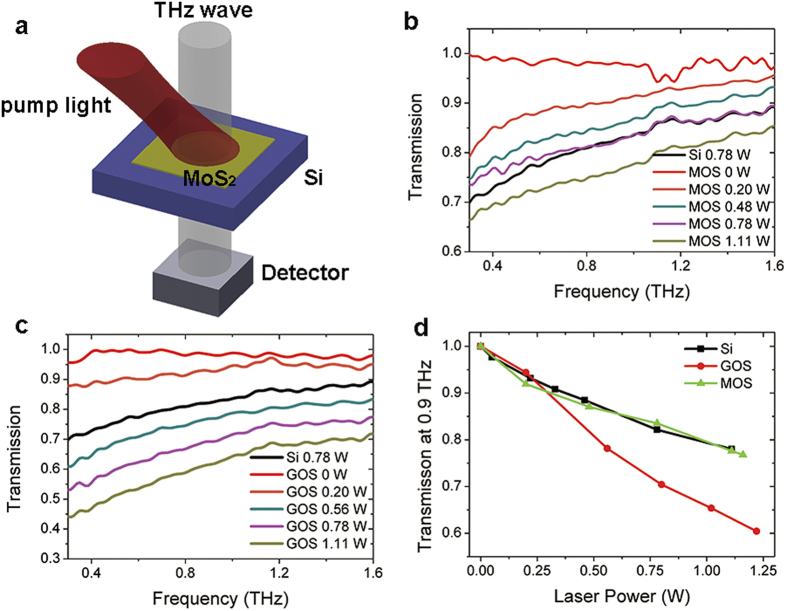
The measured THz transmissions of different samples under various optical pump powers. (**a**) A sketch map of the experiment. THz transmissions of (**b**) the unprocessed MOS sample and (**c**) the GOS sample. (**d**) Relationship between transmissions at 0.9 THz and the pumping laser power for different samples.

The measured THz transmission spectra of the MOS sample are shown in Fig. 2b. The transmittances are on the reference of the transmission spectrum of a bare silicon sample without laser illumination. It is observed that the broadband THz transmission values decrease with the pump power. This decrease is the result of the increase in conductivity of the sample caused by photogenerated carriers. So a lower transmission indicates a larger modulation of THz waves. In Fig. 2b, the transmission line of a silicon substrate is plotted (the black line) as a comparison. Under an optical pump power of 0.78 W, the MOS sample shows a bit larger transmittance compared with that of the silicon substrate. This means that MoS_2_ shows no enhancement in optical modulation of terahertz wave. Meanwhile, transmission spectra of the GOS sample under different pump powers are demonstrated in Fig. 2c. Compared with that of a silicon substrate, the transmission of GOS is much smaller when the pump power is 0.78 W. These results agree with those reported in ref. 22, demonstrating that graphene can enhance THz modulating effect to a large extent. In Fig. 2d, transmittances of different samples at 0.9 THz are plotted as a function of the optical pump power. It is illustrated that modulation depths of the MOS sample and the silicon substrate are much smaller than that of the GOS sample.

**Figure 3:**
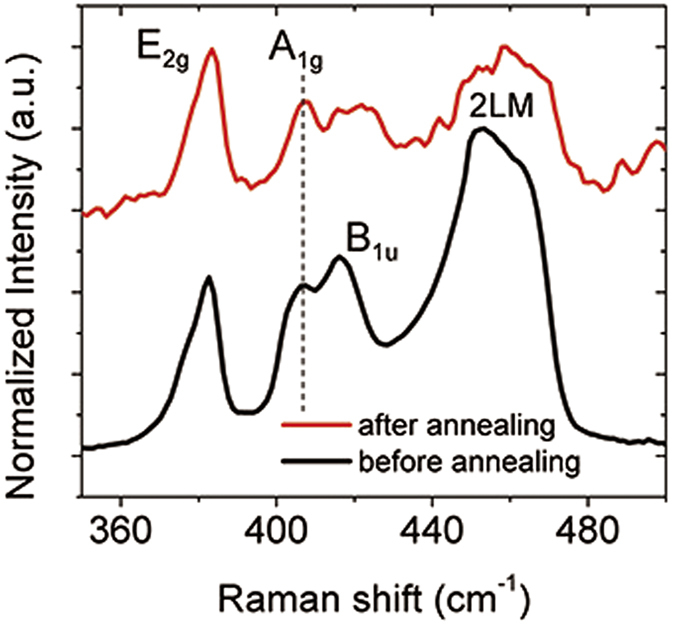
A comparison of Raman spectra of the MOS sample before and after the annealing treatment. The Raman intensity are normalized to their maxima.

It has been reported that oxygen-induced hole doping effectively enhanced the photo-luminescence of MoS_2_ and changed its electronic properties^36^,^38^. By annealing in air at proper temperature, the MoS_2_ sample can be effectively doped with oxygen and provide numerous holes. In our following experiment, the MOS sample was placed in air and annealed at 300 °C. The MOS sample after annealing treatment was characterized with Raman spectroscopy using 632 nm laser line. As shown in Fig. 3, the *E*^*1*^_*2g*_ peak lies at 383.3 cm^−1^ and the *A*_1g_ peak lies at 407.9 cm^−1^ (the red line). The Raman spectra of the MOS sample before annealing is plotted in black as a comparison. A slight blueshift of 1.3 cm^−1^ of the *A*_1g_ peak is observed compared to the sample before annealing, indicating that the sample has been p-doped.

**Figure 4:**
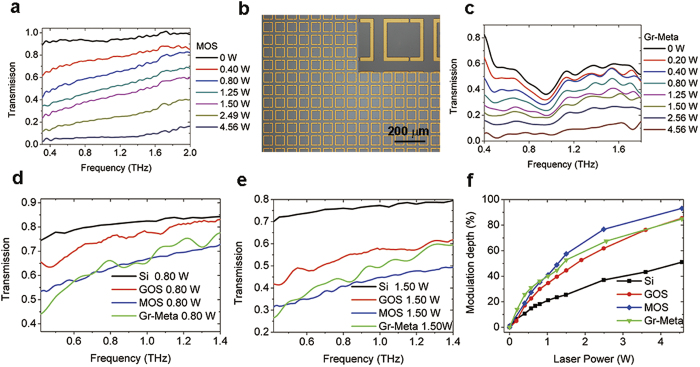
Broadband THz modulation in transmission of various samples under different optical pump powers. THz transmissions of (**a**) the annealed MOS sample and (**c**) the graphene on metamaterials sample. (**b**) Microscopic images of the THz metamaterial sample. (**d,e**) Comparisons of THz relative transmissions for different samples under the same pump power. (**f**) Modulation depths in transmission at 0.9 THz of different samples versus pumping laser power.

To figure out the effect of annealing treatment for MoS_2_, THz transmission spectra of the annealed MOS sample, together with the GOS sample as a comparison, are measured. In the measurement, the same 808 nm CW laser was used to modulate photo-generated carrier densities of these samples. The results are shown in Fig. 4. The MOS sample, which was heated in air for 0.5 h, demonstrates good optical modulating effect at a broad frequency band, presenting a transmission decline of Δ*T*_*M*_ = *T*_*0*_ − *T*_*P*_ = 87% in transmission at 0.9 THz under a large pump power of 4.56 W (here *T*_*0*_ and *T*_*P*_ is THz transmission under pump power 0 W and *P*, respectively). Under a low pump power of 0.80 W, Δ*T*_*M*_ is about 33% at 0.9 THz. This is much larger than that of the unprocessed MOS sample, whose transmission decrease is only 16.6% under the same pump power. The modulation efficiency of the MOS sample almost doubled after the annealing treatment.

Devices based on graphene-metamaterial have been comprehensively applied in THz modulators. In our experiment, a sample of THz metamaterial made of gold was fabricated, as shown in Fig. 4b. Then a layer of graphene was transferred onto this sample and covered the metamaterial. Optically modulated THz transmission spectra of this graphene-metamaterial (Gr-Meta) sample were measured as a comparison for the GOS and the MOS samples. As shown in Fig. 4c, the Gr-Meta sample demonstrates a transmission dip at ~1THz. Under a pump power of 4.56 W, the spectra demonstrate a transmission decrease from 39% to 5.8% at 0.9 THz. In Fig. 4d, transmission spectra under a pump power of 0.80 W of high-resistivity silicon, GOS, annealed MOS and Gr-Meta samples were compared. All the spectral results are divided by their corresponding transmission spectra without optical pumping, i.e., *T*_*P*=*0.8w*_/*T*_*P*=*0w*_. It is clearly demonstrated that the modulation efficiency of the annealed MOS sample is much higher than that of a silicon substrate, and even higher than that of the GOS sample. This is in sharp contrast with the results shown in Fig. 2d, as the MOS sample before annealing shows much worse modulation effect than the GOS sample. The Gr-Meta sample demonstrates higher modulation efficiency than the GOS sample, and is about the same level of the MOS sample. At lower frequencies, the Gr-Meta sample has higher modulation efficiency but at higher frequencies, the MOS sample works better. The transmission spectra of different samples under a larger pump power of 1.5 W are also compared in Fig. 4e. It can be found that the MOS sample shows higher modulation efficiency than the Gr-Meta sample, especially at higher frequencies and under a higher pump power.

To give a better view of the results, modulation depths of THz transmission at 0.9 THz of different samples are compared in Fig. 4f. The modulation depth is calculated as *M* = (*T*_0_ − *T*_P_)/*T*_0_, where *T*_0_ is the transmission without optical pumping and *T*_P_ is the transmission with pump power *P*. Under a relatively small pump power of 0.57 W, modulation depths are 14.7%, 22.6%, 27.3% and 31% for pure silicon, GOS, MOS and Gr-Meta, respectively. The modulation depth of MOS is nearly twice that of silicon. It can be seen that when the laser power is lower than 1W, the modulation depth of MOS is smaller than that of Gr-Meta; nevertheless, when the laser power is higher than 1W, the result is opposite. Under a pump power of 1.5 W, modulation depths for pure silicon, GOS, MOS and Gr-Meta are 25.4%, 44.5%, 57.5%, 52.7% respectively. Compared with pure silicon, modulation efficiencies of GOS, MOS and Gr-Meta have enhanced 75%, 126.4% and 107.5%, respectively. The modulation depths of these samples do not increase linearly with the laser power. At a large power of 4.56W, the modulation depths are 93.1% for MOS, 85.4% for GOS and 84.8% for Gr-Meta.

**Figure 5:**
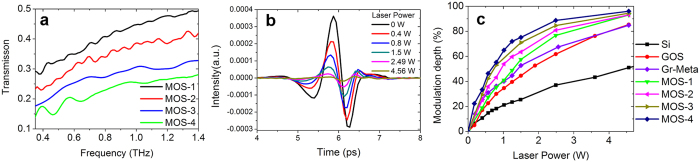
(**a**) THz spectra of the MOS sample with different annealing time, measured under an optical pump power of 1.5 W. (**b**) The time domain intensity of the transmission THz signal of the MOS-4 sample under different optical pump powers. (**c**) The relationship between modulation depths at 0.9 THz and the laser power for different samples.

As the annealing treatment significantly improves the property of MOS, it is expected that the modulation efficiency of MOS will be higher with longer annealing time. Therefore we treated the same sample of MOS with different annealing time and measured corresponding THz spectra modulated by the 808 nm CW laser. Results are shown in Fig. 5. The legends MOS-1, MOS-2, MOS-3 and MOS-4 represent MOS annealed in air at 300 °C for 0.5 h, 1.2 h, 3 h and 5 h respectively. As can be seen in Fig. 5a, transmission of MOS-4 is much smaller than that of MOS-1, which indicates that MOS-4 has a higher THz modulation efficiency. Under a large optical pump power, the transmission THz signal of MOS-4 almost vanishes (Fig. 5b). It is clearly demonstrated that the modulation depth of MOS increases with the annealing time in Fig. 5c. Under a pump power of 1 W, modulation depths at 0.9 THz for MOS-1, MOS-2, MOS-3 and MOS-4 are about 41.0%, 53.9%, 59.2% and 64.9%, respectively. An increase of 23.9% in modulation depth is achieved by treating the MOS sample with longer annealing time. Under the pump power of 1 W, modulation depths are 21.2% for silicon, 34.6% for GOS and 40.1% for Gr-Meta samples. So the modulation efficiency of MOS-4 is 3.07 times as high as that of pure silicon and 88% larger than that of GOS. It is also much larger than that of Gr-Meta (62% larger). When the pump power is as large as 4.56 W, modulation depths of MOS with different annealing time are close to 100% and approach to each other. For GOS and Gr-Meta samples, their modulation depths also reach the same point under a pump power of 4.56 W.

At lower frequencies, the THz modulation depth of MOS is also much larger than other samples. The relationship between modulation depths at 0.5 THz and the laser power is shown in Supplementary Information Fig. S1. Beside these samples above, we tried to combine annealed MoS_2_ with metamaterials to give a larger modulation depth. However, the sample with annealed MoS_2_ on metamaterials does not show any enhancement in THz modulation efficiency (see Supplementary Information Fig. S2). Additionally, the high-resistivity silicon sample with few-layer graphene covering on it (FLGOS) was also prepared and measured. FLGOS demonstrates lower THz modulation efficiency compared with GOS (see Supplementary Information Fig. S2).

## Discussion

When the electric conductivity of a material increases, this material will absorb and reflect more THz waves, and its THz transmission will decrease. The conductivity of a material can be either tuned by an external voltage signal or exerting an optical pump signal to excite photo-generated carriers in silicon. In previous studies, the mechanism of a THz wave modulator based on optically tuned graphene-silicon structure was discussed in detail^22^. The modulation depth was enhanced by graphene compared with a pure silicon substrate. This effect has been attributed to considerable carriers diffusing from silicon into the graphene layer. Due to the higher mobility of graphene compared with silicon, these carriers in graphene layer result in a larger change of conductivity than that in pure silicon. This enhancement is verified in our experiments, as the GOS sample shows a larger modulation depth in transmission than the bare silicon substrate (Fig. 4d). However, for the MOS sample, the situation is different and this theory cannot explain the modulation enhancement, as the mobility of MoS_2_ is as low as several cm/V·s^38^, much smaller than that of silicon. As a result, in contrary to what happens in the GOS sample, when charge carriers diffuse from silicon into the MoS_2_ layer, they experience a mobility decline and may results in a drop of modulation depth. This is coincident with the experiment result shown in Fig. 2b.

**Figure 6:**
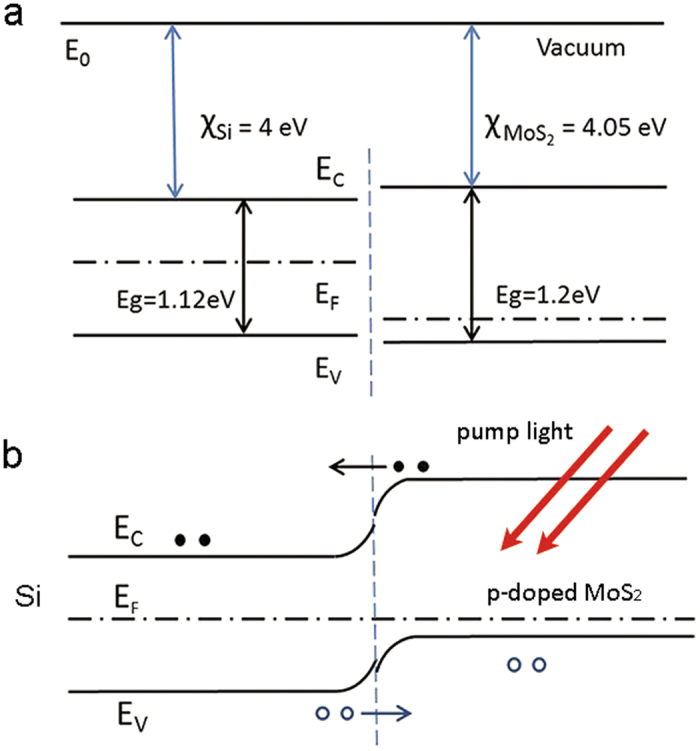
The band schemes of MoS_2_-silicon system. (**a**) Energy bands of MoS_2_ and silicon before they contact. (**b**) Band bending after MoS_2_ and silicon contact and the flowing of photogenerated carriers.

It has been shown above that MoS_2_ is p-doped after annealed in air. In this process the impurity concentration increases so the mobility of MoS_2_ is expected to decrease^38^. Meanwhile, the hole concentration of MoS_2_ becomes larger and this makes the Fermi level energy moving to the valence band maximum. The change in energy band structure affects the way MoS_2_ contacts with the silicon substrate. In the MOS sample without annealing treatment, both silicon and MoS_2_ are n-doped, so the barrier at the silicon-MoS_2_ interface is rather small and the corresponding built-in electric field is small. In contrast, in the annealed MOS sample, silicon and MoS_2_ compose an n-p type heterostructure and the barrier at the silicon-MoS_2_ is relatively large. Silicon and MoS_2_ have electron affinities and energy band gaps with only small differences. The conduction band difference is 0.05 eV and the valence band difference is 0.03 eV. The band structure schemes of silicon-MoS_2_ system are depicted in Fig. 6. The resistivity of the slightly n-doped silicon substrate is more than 5000 Ω· cm, therefore it can be interfered that the electric concentration in silicon is less than 10^13^ cm^−3^. The Fermi level energy *E*_F_ is about 0.06 eV upon the center of bandgap in silicon. In p-doped MoS_2_, *E*_F_ is close to the valence band maximum. Hence the Fermi level energy in silicon is higher than that in MoS_2_. When these two material contacts, electrons flow from silicon to MoS_2_ and holes flow in the opposite direction. At equilibrium, the Fermi level energy is united and the built-in electric field is orientated from silicon to MoS_2_. Due to the mismatch between the crystal lattices of silicon and MoS_2_, there exist surface states at the interface of MoS_2_, then *E*_F_ is pinned about 1/3 *E*_g_ (0.4 eV) upon the valence band maximum, as shown in Fig. 6b.

When a pumping beam illuminates the MOS sample, nonequilibrium photogenerated carriers are excited both in silicon and in MoS_2_. As MoS_2_ layer is rather thin, it only absorbs a small part of pumping light. Most light are absorbed by silicon and most free charge carriers are generated in it. Moreover, nonequilibrium carriers in MoS_2_ bear a rather low mobility, and only make a relatively small contribution to the change of the conductivity. Thus the conductivity change of the silicon-MoS_2_ system is mainly determined by the generated free carriers in silicon, who gives an extra photoconductance to silicon. The photoconductance of silicon can be expressed as:


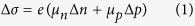


Here *e* is the elementary charge, *μ*_*n*_ and *μ*_*p*_ are charge mobilities of electrons and holes in silicon, respectively. Δ*n* and Δ*p* are concentrations of nonequilibrium electrons and holes generated by pump light. In slightly doped silicon, such as high-resistivity silicon used in our experiment, *μ*_*n*_ = 1450 cm^2^/V·s and *μ*_*p*_ = 500 cm/V·s.

Due to the built-in electric field, electrons diffuse from MoS_2_ to silicon, and holes in silicon move to MoS_2_. This makes Δ*n* larger and Δ*p* smaller in silicon compared with the case of pure silicon. As the electron mobility *μ*_*n*_ is nearly three times that of hole mobility *μ*_*p*_, free electrons play a more important role in change of the photoconductance Δ*σ* than holes. Therefore, the increase in Δ*n* gives rise to a larger Δ*σ*, which results in lower THz transmission and a larger THz modulation depth. In addition, the p-doped MoS_2_ has an effect similar with a minority carrier trap for silicon. In the slightly n-doped silicon, the number of photogenerated carriers is much larger than the intrinsic carriers. So the recombination probability and the lifetime *τ* of nonequilibrium electrons is mainly determined by Δ*p* and *τ* is inversely proportional to Δ*p*. The electronic-hole pairs are separated by the built-in electric field and a larger number of holes are trapped in MoS_2_. The decrease of hole concentration Δ*p* depresses the recombination probability of free electrons and holes in silicon and consequently increases the lifetime of free electrons. This leads to a larger Δ*n* and an increase of the photoconductance of silicon. Hence an enhancement of THz modulation is observed in the annealed MOS sample. Our theory agrees well with results in Fig. 5. As the annealing time of MOS is increased, the Fermi level energy in MoS_2_ is closer to the valence band and the built-in electric field becomes stronger. Therefore, under optical pumping, more electrons flow to silicon and more holes move from silicon to MoS_2_. This leads to a larger enhancement of THz modulation efficiency for MOS.

We define the number of photogenerated electron-hole pairs per second and per cm^2^ as *N*_*g*_, then


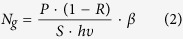


Herein, *P* is the laser power, *R* is reflection of pump light reflected from the sample, S is size of the illuminated area, *h*υ is the photon energy of pump light and *β* is the percentage of photons that effectively generate electron-hole pairs in silicon. When *P* = 1 W, *N*_*g*_ can be estimated to be about 5 × 10^17^ s^−1^·cm^−2^.

Therefore the average concentration of generated carriers in the conducting silicon layer can be approximatively described as *n*_*g*_ = *p*_*g*_ = *N*_*g*_*τ*_g_/*d*, where *τ*_g_ is the lifetime of nonequilibrium carriers (the value of *τ*_g_ is assumed to be about 10 *μ*s) and *d* is the thickness of photogenerated conducting layer of silicon. According to equation (1), the conductivity of silicon under pump power *P* = 1 W is *σ*_*g*_ = *n*_*g*_(*μ*_*n*_ + *μ*_*p*_)*e* = 3 Ω^−1^·cm^−1^, which is about 10^4^ times larger than conductivity of the high-resistivity silicon without photodoping.

**Figure 7:**
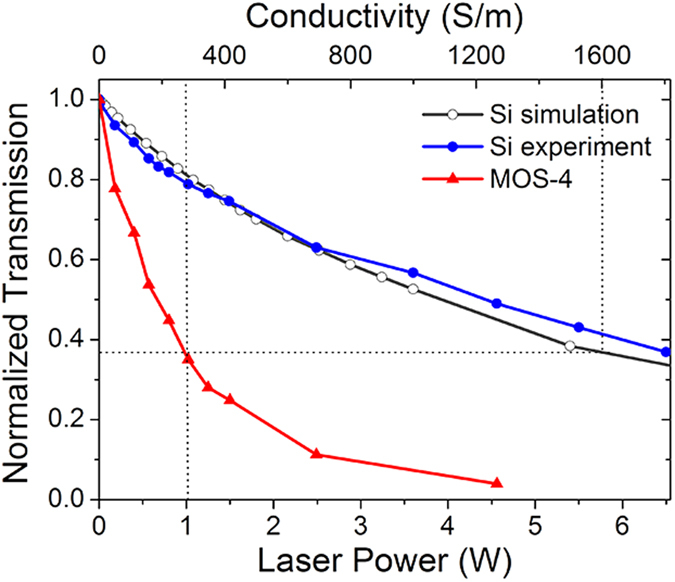
Simulated and measured transmissions (at 0.9 THz) of silicon substrate under different optical pump power, with experiment results of MOS-4 as a comparison. The top axis is the conductivity of the photogenerated conducting layer in silicon corresponding to the laser power.

As the conductivity of silicon is proportional to the pump power, we can equivalently replace the pump light with the change in conductivity of silicon and carry out a simulation. In the simulation, THz waves ranging from 0.2–2.2 THz transmit a silicon wafer. The thickness of the photogenerated conducting layer of silicon is set to be 5 *μ*m^22^. The calculated relationship between THz transmission and conductivity of silicon is shown in Fig. 7.

For MOS, due to the effect of MoS_2_ layer, the electron concentration *n*_*g*_ increases while the hole concentration *p*_*g*_ decreases in silicon. Compared with pure silicon, the photoconductivity of MOS is 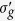 = *σ*_*g*_ + Δ*σ*_*g*_ = *σ*_*g*_ + Δ*σ*_*1*_ + Δ*σ*_*2*_. Here Δ*σ*_*g*_ is the conductivity change caused by annealed MoS_2_, Δ*σ*_*1*_ is the change caused by injection of electrons from MoS_2_ and decrease of *p*_*g*_, and Δ*σ*_*2*_ is resulted by increase of *n*_*g*_ due to a longer lifetime of free electrons. However, as the number of electrons injected from MoS_2_ is much less than *n*_*g*_ and the mobility of holes in silicon is small, Δ*σ*_*1*_ is relatively small compared with Δ*σ*_*2*_. Thus the conductivity change of the device is mainly dominated by Δ*σ*_*2*_. As mentioned above, *n*_*g*_ = *N*_*g*_*τ*_g_/*d*, and *τ*_g_ is proportional to 1/*p*_*g*_. When holes in silicon flow to MoS_2_, if *p*_*g*_ decreases by about 80%, *n*_*g*_ will increases over 4 times and 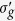 ≈ 5*σ*_*g*_ = 15 Ω^−1^ cm^−1^. Under a pump power *P* = 1 W, MOS-4 presents a THz transmission of 0.37 at 0.9 THz. This corresponds to a conductivity of about 16 Ω^−1^ cm^−1^ according to our simulation model for silicon, about 5 times of *σ*_*g*_, indicating that MoS_2_ traps about 80% of generated holes in silicon.

In conclusion, we experimentally demonstrated an effective and broadband THz wave modulator based on optically tuned multilayer MoS_2_ on silicon. With an annealing treatment in air, the MoS_2_ was p-doped and showed dramatic changes in electrical and optical properties. The THz modulation depth at 0.9 THz increased from 16.6% to 33% after a preliminary annealing process with an optical pump power of 0.80 W. Further experiment results show that the THz modulation efficiency of MOS becomes much larger with longer annealing time. It exhibited a modulation depth of 64.9% at 0.9 THz under a pump power of 1 W, which is more than three times as larger as that of pure silicon. Under a larger pump power of 4.56 W, this depth is about 96%. Optically tuned THz modulators based on graphene-silicon and graphene-metamaterials were also fabricated and measured as comparisons. Results show that MOS annealed for 5 hours has a modulation efficiency 88% larger than that of GOS and 62% larger than Gr-Meta. Then we proposed an analytical model to explain this enhancement of THz modulation efficiency in annealed MOS. In our theory, MoS_2_ traps much of photogenerated holes from the silicon substrate, which leads to a decrease in lifetime of free electrons and gives rise to an increase of electron concentration and conductivity of silicon. The numerically calculated THz transmission spectra agree well with experimental results. These results demonstrate that MoS_2_ is a promising material to be applied in THz modulators with high efficiency.

## Methods

### Sample preparation

The 1 cm × 1 cm multilayer MoS_2_ film is grown on a SiO_2_/Si sample. A layer of poly-methyl methacrylate (PMMA) was coated onto the sample. Then the SiO_2_ layer was etched by the NaOH solution (~2 mol/L) and transferred to the high-resistivity silicon substrate whose size is 1.5 cm × 1.5 cm. During the annealing treatment of MoS_2_, the heating rate is ~5 °C/min. The ambient temperature stayed at 300 °C for a time, and then the sample was cooled with a rate of ~1.25 °C/min. Samples were measured with protection of N_2_ gas.

### Theoretical and numerical calculation

Parameter values used in equation (2) are *R* = 0.36. *S* = 1.76 cm^2^, *hν* = 1.54 eV, *β* = 0.3. The thickness of photogenerated conducting layer in silicon is d = 5 *μ*m. The value of carrier’s lifetime *τ*_g_ ≈ 10 *μ*s. The numerical simulation was performed with a finite different time domain software (Lumerical FDTD Solutions). In the simulation, the conductivity of the conducting silicon layer varied from 0.02 S/m to 2000 S/m. The conductivity of silicon corresponding to a pump power of 1 W was set to be 300 S/m.

## Additional Information

**How to cite this article**: Cao, Y. *et al*. Optically tuned terahertz modulator based on annealed multilayer MoS_2_. *Sci. Rep.* **6**, 22899; doi: 10.1038/srep22899 (2016).

## Supplementary Material

Supplementary Information
