# Supplementary material for: Optically tuned terahertz modulator based on annealed multilayer MoS2
Source: Sci Rep. 2016 Mar 8;6:22899. doi: 10.1038/srep22899 (PMC4782166; doi:10.1038/srep22899)
Supplement: Supplementary Information [file srep22899-s1.pdf]

Supplementary Information

Optically tuned terahertz modulator based on annealed multilayer MoS_2

Yapeng Cao[1, 2], Sheng Gan[3], Zhaoxin Geng[1,4※], Jian Liu[2※], Yuping Yang[5], Qiaoling Bao[3] and Hongda Chen[1※]

[1] State Key Laboratory of Integrated Optoelectronics, Institute of Semiconductors, Chinese Academy of Sciences, Beijing, 100083, China

[2] State Key Laboratory of Superlattices and Microstructures, Institute of Semiconductors, Chinese Academy of Sciences, Beijing, 100083, China

[3] Institute of Functional Nano and Soft Materials (FUNSOM), Soochow University, Suzhou 215123, China

[4] School of Information Engineering, Minzu University of China, Beijing 100081, China

[5] College of Science, Minzu University of China, Beijing 100081, China

Modulation depths vs the optical pump power at 0.5 THz

The relationship between modulation depths at 0.5 THz and the laser power for different samples is shown in Fig. S1. Under the pump power of 1W, modulation depths at 0.5 THz are 22.0% for silicon, 44.3% for GOS, 51.2% for Gr-Meta, 50.8% for MOS-1, 64.3% for MOS-2, 68.8% for MOS-3 and 74.0% for MOS-4. We define the index N_m of a sample as how many times its modulation efficiency is as large as that of pure silicon. Then for silicon, N_m is 1 and for MOS-4 N_m is 3.36. As for GOS and Gr-Meta, the values of N_m is 2.01 and 2.33. It can be seen that the modulation efficiency of MOS-4 is much higher than that of GOS and Gr-Meta. The modulation depth of MOS-4 reaches 50% under a pump power of 0.47 W and 80% under a pump power of 1.20 W.

Modulation depths of MOS-Meta and FLGOS

The sample with annealed MoS_2 covering on metamaterials (MoS_2-Meta) was fabricated. Its substrate is high-resistivity silicon. Additionally, the sample with few-layer (3~10 layers) graphene covering on high-resistivity silicon (FLGOS) was also prepared. Optically tuned THz spectra of these two samples were measured and compared with other samples. Modulation depths at 0.9 THz under different optical pump powers are shown in Fig. S2.

The combination of graphene and metamaterials gives rise to an enhancement of the modulation efficiency compared with GOS. However, for MoS_2-Meta, its modulation effect is rather bad, even worse than the silicon substrate. In the Gr-Meta sample, the extra conductivity change leading to modulation enhancement happens in the graphene layer, which is on the surface of the sample and is rather thin. Therefore, as metamaterials enhance the local field at surface of the sample, the modulation enhancement is magnified. For the MoS_2-based sample, the extra conductivity change and modulation enhancement originate from the change of carrier density in silicon, instead of in MoS_2. The enhanced local surface field does not contribute to this change. On the contrary, metamaterials may block the contact of silicon and MoS_2. Gold metamaterials may also act as recombination center for electrons and holes,

which prevents MoS_2 from trapping holes from silicon and makes the modulation enhancement caused by MoS_2 extinguishes.

For the FLGOS sample, its modulation efficiency is higher than silicon, but lower than GOS. As is known, modulators based on graphene rely on the high mobility of graphene. The unsatisfactory modulation effect of FLGOS is likely due to the fact that few-layer graphene has a lower mobility compared with single-layer graphene.

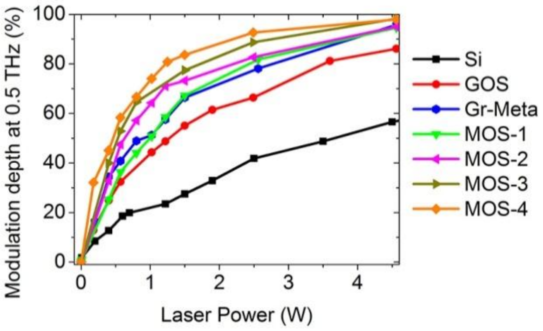

Figure S1. The relationship between modulation depths at 0.5 THz and the laser power for different samples. MOS-1, MOS-2, MOS-3, MOS-3 and MOS-4 represent MOS samples annealed in air at 300 ℃ for 0.5 h, 1.2 h, 3 h and 5 h respectively.

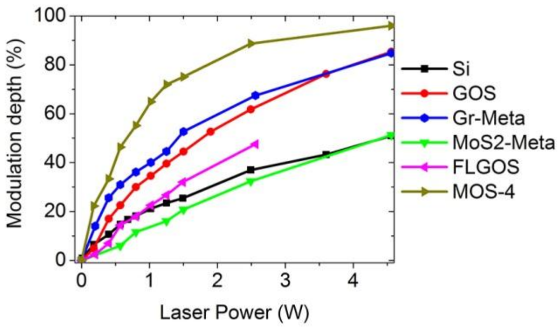

Figure S2. The relationship between modulation depths at 0.9 THz and the laser power for different samples.
